# Long-Term Per- and Polyfluoroalkyl Substances Exposure and Kidney Function in Taiwanese Adolescents and Young Adults: A 10-Year Prospective Cohort Study

**DOI:** 10.3390/jox16010016

**Published:** 2026-01-21

**Authors:** Chien-Yu Lin, Hui-Ling Lee, Ta-Chen Su

**Affiliations:** 1 Department of Internal Medicine, En Chu Kong Hospital, New Taipei City 237, Taiwan; 00724@km.eck.org.tw; 2 School of Medicine, Fu Jen Catholic University, New Taipei City 242, Taiwan; 3 Department of Chemistry, Fu Jen Catholic University, New Taipei City 242, Taiwan; 076308@mail.fju.edu.tw; 4 Department of Internal Medicine, Tungs’ Taichung MetroHarbor Hospital, No. 699, Sec. 8, Taiwan Blvd., Wuqi District, Taichung 435, Taiwan; 5 Department of Internal Medicine, National Taiwan University Hospital, Taipei 100, Taiwan; 6 Institute of Environmental and Occupational Health Sciences, National Taiwan University, Taipei 100, Taiwan

**Keywords:** PFAS (per- and polyfluoroalkyl substances), kidney function, eGFR (estimated glomerular filtration rate), longitudinal study, young population, Taiwan

## Abstract

**Background and hypothesis**: Per- and polyfluoroalkyl substances (PFAS) are highly persistent synthetic chemicals that can accumulate in renal tissue and potentially disrupt kidney function. Most prospective studies on PFAS–renal associations have focused on middle-aged or older adults, leaving uncertainty about whether similar patterns exist in younger populations. **Methods**: We investigated decade-long trajectories of plasma concentrations of 11 PFAS and their longitudinal associations with estimated glomerular filtration rate (eGFR) among 529 Taiwanese adolescents and young adults (aged 12–30 years) enrolled in the prospective YOung TAiwanese Cohort (YOTA), with measurements obtained in 2006–2008 and 2017–2019. **Results**: Nearly all plasma PFAS declined significantly over the 10-year period. Despite these reductions, higher baseline levels and greater annualized increases (Δln-PFAS/Δt) in linear perfluorooctanoic acid (PFOA), linear and branched perfluorooctane sulfonic acid (PFOS), perfluorononanoic acid (PFNA), and perfluorodecanoic acid (PFDA) were consistently associated with larger eGFR gains over time (β = 0.33–0.40, q < 0.05). In complementary models using follow-up eGFR as the outcome, both baseline and cumulative PFAS changes (Δln-PFAS) remained positively associated with higher eGFR (β = 1.71–3.84, q < 0.05). Polynomial analyses further indicated mild non-linear exposure–response patterns for several PFAS, suggesting that renal effects may deviate from linearity across exposure ranges. The composite PFAS exposure index (mean of standardized ln-PFAS concentrations) was robustly associated with higher eGFR across sensitivity analyses excluding participants with chronic conditions. These associations were more pronounced among individuals with greater metabolic or physiological vulnerability. **Conclusions**: Higher PFAS exposure was associated with elevated eGFR in young adults, which may be consistent with early glomerular hyperfiltration or other renal hemodynamic alterations. These findings raise the hypothesis of early renal stress in early life and underscore the need for ongoing biomonitoring and longitudinal follow-up with additional kidney injury markers to clarify long-term renal consequences.

## 1. Introduction

Since the 1950s, per- and polyfluoroalkyl substances (PFAS) have been used across consumer and industrial applications, leading to global ubiquity due to their extreme persistence and mobility [1]. Diet, especially fish and seafood, often represents a major exposure route, alongside drinking water, packaging, indoor dust, and air [2]. Recent reviews continue to link PFAS body burdens with adverse metabolic, immunologic, and endocrine outcomes [3]. Globally, PFAS regulation has intensified over the past two decades. The European Union has enforced manufacturing and use restrictions under the REACH framework, while in the United States, the Environmental Protection Agency introduced the first national drinking-water standards for six PFAS compounds in 2024, alongside updated guidance for PFAS waste treatment and disposal [4,5]. Through coordinated global actions and regulatory interventions, human exposure to PFAS has shown a gradual decline in recent years [6]. In Taiwan, PFAS contamination from high-tech manufacturing has been reported in river systems for over a decade [7,8]. In response, growing public awareness has led semiconductor manufacturers to develop fluorine-free or low-PFAS alternatives in photoresists and surfactants [9]. Similar to global patterns, our recent longitudinal studies in Taiwanese cohorts show measurable serum PFAS burdens and decade-long declines in several legacy compounds, with cardiometabolic associations in younger populations [10,11]. Despite stricter global regulations, the complete elimination of PFAS remains challenging because effective fluorine-free replacements are scarce [12]. As a result, background exposure persists, and emerging evidence shows that even chronic, low-level PFAS exposure can be linked to adverse health effects and may exacerbate health inequities among vulnerable populations [13,14]. These findings highlight the continued need for robust regulatory action and innovation in safer chemical alternatives.

Experimental studies have demonstrated that PFAS can directly influence renal structure and physiology through several mechanisms. In animal and in vitro models, long-chain PFAS such as perfluorooctanoic acid (PFOA) and perfluorooctane sulfonic acid (PFOS) accumulate in the proximal tubule, where they induce oxidative stress, mitochondrial dysfunction, and apoptosis of tubular epithelial cells [15,16]. PFAS exposure has also been shown to alter the expression of organic anion transporters, impair fatty-acid β-oxidation, and activate peroxisome proliferator-activated receptor-α (PPARα) signaling, leading to renal hypertrophy and dysregulated lipid metabolism [17,18]. Growing epidemiological evidence links PFAS exposure to diverse renal disorders. A recent meta-analysis reported a modest but significant increase in kidney cancer risk among populations with high PFAS exposure [19]. Beyond cancer, studies have focused on PFAS and kidney function, particularly estimated glomerular filtration rate (eGFR). Cross-sectional studies in adults and adolescents consistently show that higher serum PFAS concentrations, particularly PFOA, PFOS, and perfluorononanoic acid (PFNA), are associated with lower eGFR, indicating reduced kidney function [20,21,22,23]. In contrast, certain compounds such as perfluorohexane sulfonic acid (PFHxS) have shown positive or null associations [22]. Longitudinal findings remain mixed. Participants in the Fernald Community Cohort (median age ≈ 38 years) were followed for up to 18 years (median ≈ 13 years). Higher PFNA, PFHxS, and perfluorodecanoic acid (PFDA) levels were associated with lower eGFR, whereas methyl-perfluorooctane sulfonamide showed a positive association [24]. In a Taiwanese cohort (mean age ≈ 56 years), branched PFOS and PFHxS predicted greater 4-year eGFR decline, though some PFAS, such as PFOA, showed positive or null associations [25]. In comparison, the Swedish Prospective Investigation of the Vasculature in Uppsala Seniors (PIVUS) study (70–80 years) found that long-chain PFAS (e.g., PFNA, PFDA, Perfluoroundecanoic acid (PFUdA)) were positively associated with eGFR over 10 years, while shorter-chain PFAS showed inverse effects [26]. The authors proposed that the observed positive associations for certain long-chain PFAS may reflect early glomerular hyperfiltration, compound-specific tubular reabsorption, or non-linear exposure–response relationships, resulting in an apparent increase in eGFR driven by compensatory or toxicokinetic mechanisms rather than genuine improvement in renal function [26].

Collectively, these heterogeneous findings across studies suggest that PFAS–renal associations may vary by compound type, exposure range, and population characteristics such as age. Most longitudinal investigations have focused on middle-aged or older adults, leaving uncertainty as to whether similar or distinct PFAS–eGFR relationships exist in younger populations whose renal physiology, metabolic capacity, and exposure dynamics may differ substantially. In addition, PFAS occur as linear and branched isomers with distinct toxicokinetic profiles, including differences in protein binding and renal clearance, underscoring the relevance of isomer-specific exposure–response evaluation [27]. To address this gap, we conducted an analysis within the YOung TAiwanese Cohort (YOTA), a prospective study of adolescents and young adults, to characterize longitudinal changes in plasma PFAS concentrations and evaluate their associations with kidney function as measured by eGFR. Unlike prior studies, our analysis jointly considered both baseline PFAS levels and their annualized changes (ΔPFAS/Δt), providing a dynamic perspective on PFAS kinetics and renal responses during early adulthood, a critical developmental window that may reveal early physiological adaptations preceding clinically detectable renal impairment.

## 2. Materials and Methods

### 2.1. Study Population and Data Collection

This longitudinal study drew on data from the YOTA Cohort, which initially enrolled 886 adolescents and young adults aged 12–30 years residing in the Taipei metropolitan region between 2006 and 2008 [28,29]. A decade later, from 2017 to 2019, 542 members of the YOTA Cohort participated in a follow-up assessment designed to examine the influence of residential and dietary factors on cardiometabolic health [10,11]. All participants provided written informed consent prior to enrollment. Study protocols were approved by the Research Ethics Committee of National Taiwan University (IRB No: 9561705054 and 201604089RINA). Baseline serum PFAS concentrations were determined for all 886 participants. Among the 542 individuals who participated in the follow-up, 11 were excluded due to missing serum samples and 2 for lacking income data, leaving 529 participants in the final analysis. Participant characteristics are described in the Supplementary Materials.

### 2.2. Measurement of Plasma PFAS Concentrations

Plasma specimens collected at baseline (2006–2008) and follow-up (2017–2019) were stored at −80 °C until analysis. Quantification of plasma PFAS was performed at the Department of Chemistry, Fu Jen Catholic University, using an automated on-line solid-phase extraction liquid chromatography–tandem mass spectrometry (on-line SPE LC–MS/MS) system. The analytical platform consisted of dual high-performance liquid chromatography pumps (Agilent 1260 series, Agilent Technologies, Santa Clara, CA, USA) coupled to a triple quadrupole mass spectrometer (API 3000, Applied Biosystems/MDS SCIEX, Concord, ON, Canada) operated in negative electrospray ionization mode. The analytes included PFHxS, linear PFOS, perfluoroheptanoic acid (PFHpA), linear PFOA, PFNA, PFDA, PFUdA, perfluorododecanoic acid (PFDoA), and N-methyl-perfluorooctane sulfona-mide acetic acid (N-MeFOSAA), as well as branched PFOS and branched PFOA isomers. Limits of detection ranged from 0.002 to 0.150 ng/mL. Values below the detection limit were imputed as LOD/√2. Method accuracy was assessed by recovery, calculated as Recovery (%) = [(C_measured − C_blank)/C_spiked] × 100, and precision was evaluated using the coefficient of variation (CV% = SD/mean × 100) for replicate measurements. Detection frequencies were high across both sampling periods. In baseline samples, detection rates were 100% for all linear and branched PFOA and PFOS species and for most long-chain PFAS, with slightly lower but still high detection for PFHpA and N-MeFOSAA. Similar patterns were observed at follow-up. All laboratory analyses were conducted by personnel blinded to participant characteristics. A comprehensive description of chemicals, sample preparation, instrumental parameters, calibration procedures, limits of quantification, detection frequencies, and intra- and inter-day analytical variability is provided in the Supplementary Materials and Supplementary Tables S1 and S2.

### 2.3. Measurement of eGFR

We estimated eGFR from serum creatinine using age-appropriate equations. For participants < 18 years at baseline, eGFR was calculated with the bedside Schwartz equation (eGFR = 0.413 × height [cm]/serum creatinine [mg/dL]) [30]; for those ≥ 18 years, we used the race-free 2021 CKD-EPI creatinine equation [31]. All follow-up assessments occurred 9–12 years later, when participants were ≥18 years; therefore, follow-up eGFR was uniformly computed with CKD-EPI (creatinine). Chronic kidney disease (CKD) was defined as eGFR < 60 mL/min/1.73 m^2^ [32].

### 2.4. Anthropometric and Biochemical Data

Covariates were fixed at baseline to preserve temporal ordering and minimize reverse causation. This also avoids conditioning on variables later influenced by PFAS or early kidney changes, reducing over-adjustment/collider bias and improving comparability across models. During the examination, trained interviewers collected information on participants’ sociodemographic characteristics and lifestyle factors. Participants who reported exercising regularly in daily life were defined as having an exercise habit. Smoking behavior was categorized as current smoker, environmental tobacco smoker (ETS), or non-smoker. Monthly household income was dichotomized as ≥TWD 50,000 or <TWD 50,000. Body mass index (BMI) z-scores were calculated according to age group. Adolescents were defined as individuals younger than 20 years. For participants aged ≥ 20 years, z-scores were computed as (individual BMI − mean BMI)/standard deviation. For those aged 12–19 years, BMI z-scores were derived using the WHO AnthroPlus software [33]. Insulin resistance was assessed using the homeostasis model assessment of insulin resistance (HOMA-IR), a validated indicator for both adolescents and adults [34]. Diabetes mellitus (DM) was defined as fasting glucose ≥ 126 mg/dL or current use of insulin or oral hypoglycemic agents. Blood pressure was measured twice, and the mean value was used for classification. In adults, hypertension was defined as mean systolic/diastolic blood pressure ≥ 140/90 mmHg or current antihypertensive medication use. Among adolescents, hypertension was defined as systolic or diastolic blood pressure at or above the age-, sex-, and height-specific 95th percentile [35]. Hyperlipidemia was defined as low-density lipoprotein cholesterol (LDL-C) ≥ 130 mg/dL, triglycerides ≥ 200 mg/dL, or current treatment for dyslipidemia. Because the YOTA participants were relatively young, the prevalence of chronic metabolic diseases such as hypertension, DM, and hyperlipidemia was low. Therefore, in regression analyses, systolic blood pressure (SBP), LDL-C, and HOMA-IR were included as continuous covariates to more precisely represent cardiometabolic variation within this population.

### 2.5. Statistical Analysis

Within-person differences in PFAS between baseline (2006–2008) and follow-up (2017–2019) were assessed with paired *t* tests. Because PFAS and HOMA-IR were right-skewed, values were natural-log transformed (ln). Ln-PFAS variables were then standardized to z-scores, and a PFAS exposure index was defined as the mean of the z-standardized ln-concentrations across constituents. The z-score-based composite index is one of a suite of exposure index methods commonly applied in environmental epidemiology to summarize correlated but non-interchangeable mixture components [36]. This method has been widely implemented in studies examining the health effects of PFAS, with applications spanning metabolic, renal, and other outcomes [37,38]. In multiple linear regression analyses, the annualized change in eGFR (ΔeGFR/Δt) was modeled as the dependent variable. The baseline standardized ln-PFAS and standardized annualized change in ln-PFAS (Δln-PFAS/Δt) were simultaneously included as exposure variables. The model additionally adjusted for the following baseline covariates (Model 1): age, sex, income, exercise, smoking, BMI z-score, SBP, LDL-C, HOMA-IR, and baseline eGFR. To control for multiple comparisons, the Benjamini–Hochberg false discovery rate (FDR) procedure was applied to the Δln-PFAS/Δt and Δln-PFAS tests, and the corresponding q-values were reported. To explore potential non-linear dose–response relationships between PFAS exposure and annualized eGFR change, polynomial regression terms were examined. When quadratic (x^2^) or cubic (x^3^) terms reached statistical significance, the corresponding fitted trend (quadratic or cubic) was used for graphical presentation. Scatterplots were generated using model-predicted values from multivariable-adjusted regression models to illustrate the exposure–response patterns. Regression coefficients and significance levels were derived from the same multivariable-adjusted models controlling for covariates defined in Model 1. Subgroup analyses were conducted, and interaction terms between each subgroup variable and the PFAS exposure index were added to the full model to test for statistical interaction (P for interaction).

In sensitivity analyses, we evaluated the robustness of the findings by sequentially re-estimating the multivariable models after excluding participants with CKD, DM, hypertension, hyperlipidemia, and adolescents, respectively. To further validate the associations, we constructed an additional model that used follow-up eGFR as the dependent variable and included both baseline ln-transformed PFAS concentrations and their changes (Δln-PFAS) as exposures, while adjusting for Model 1 covariates and follow-up duration. Moreover, to account for within-person correlations over time, linear mixed-effects models were fitted with repeated eGFR measurements as the outcome, specifying a random intercept and fixed effects for time (years since baseline; baseline = 0), baseline ln-PFAS (per 1 SD), and their interaction term (time × ln-PFAS) to examine potential modification of eGFR trajectories. Covariates included in these mixed-effects models were identical to those in Model 1, except for baseline eGFR, which was excluded since eGFR at time 0 served as the outcome variable. All statistical analyses were performed using IBM SPSS Statistics, version 30.0 (IBM Corp., Armonk, NY, USA). Statistical significance was defined as a two-sided *p* -value or FDR-adjusted q-value less than 0.05.

## 3. Results

Table 1 presents the baseline characteristics of the 529 participants assessed in 2006–2008 and during follow-up in 2017–2019. The proportion of individuals reporting regular exercise increased markedly from 30.6% to 53.7%, while the prevalence of active smoking rose slightly from 12.1% to 15.1%. Over the follow-up period, the prevalence of hyperlipidemia almost doubled (from 16.4% to 29.9%), whereas DM (1.3% to 3.2%) and hypertension (6.2% to 6.4%) showed smaller increases. The prevalence of CKD remained low and stable. In terms of continuous variables, both BMI and SBP increased over time. Similarly, LDL-C, HOMA-IR, and eGFR showed clear elevations. As shown in Table 2, plasma PFAS concentrations exhibited compound-specific temporal patterns between 2006–2008 and 2017–2019. Most PFAS declined over the 10-year follow-up, with geometric mean reductions ranging from approximately 20% to 80%, indicating substantial within-person decreases. These declines were most pronounced for branched PFOS, PFDA, PFUdA, and N-MeFOSAA. In contrast, branched PFOA showed a modest increase, while linear PFOS and PFHxS remained relatively stable over time.

**Figure 1:**
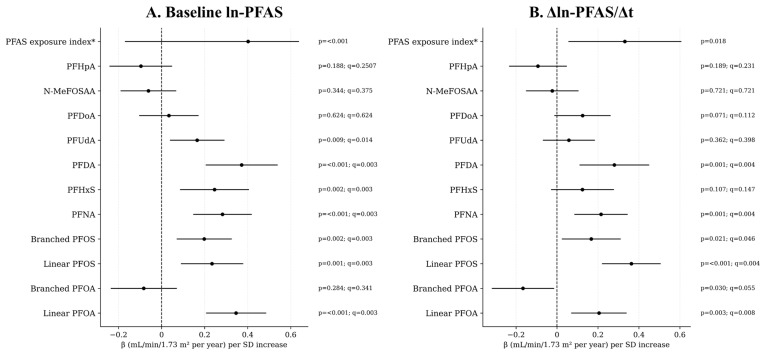
Annualized eGFR change per 1-SD increase in standardized ln-PFAS. Panels show β estimates and 95% CI from multivariable linear models for (**A**) baseline ln-PFAS and (**B**) annualized change (Δln-PFAS/Δt). Nominal *p*-values and FDR-adjusted q-values are shown to the right. All models are adjusted for covariates defined in Model 1 (see text). * The PFAS exposure index is the average of standardized (z-score transformed) PFAS concentrations.

**Figure 2:**
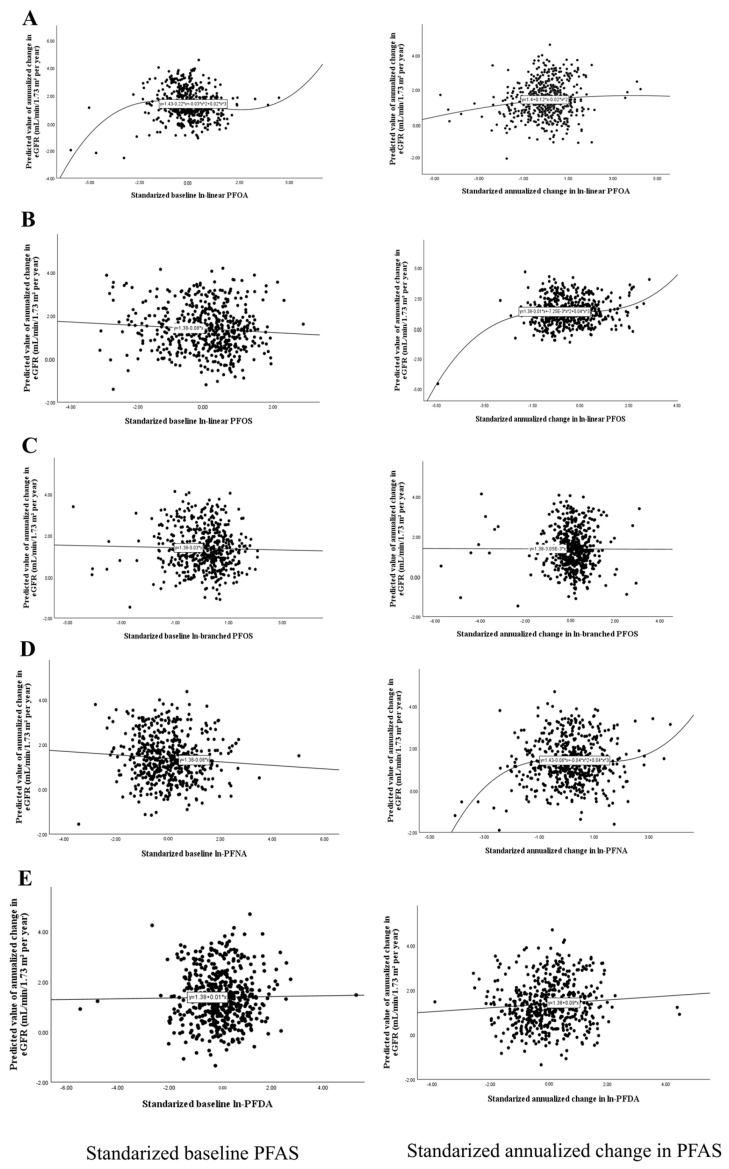
Scatterplots showing the relationships between standardized PFAS measures and annualized eGFR change. Fitted trends (linear, quadratic, or cubic) were selected according to the significance of polynomial terms in regression models. (**A**) Linear PFOA, (**B**) linear PFOS, (**C**) branched PFOS, (**D**) PFNA, and (**E**) PFDA. Scatterplots display model-predicted values and are provided for illustration; statistical inference is based on regression estimates in Figure 1.

Figure 1 summarizes the associations between baseline and annualized changes in PFAS concentrations (per 1-SD increase in standardized ln-PFAS) and the annualized eGFR change after multivariable adjustment. Several PFAS were positively associated with greater increases in eGFR over time. Specifically, linear PFOA, linear PFOS, branched PFOS, PFNA, and PFDA showed significant positive associations for both baseline and Δln-PFAS/Δt measures (all q < 0.05). PFHxS and PFUdA displayed a weaker yet statistically significant baseline association, but their change over time was not significant. Other PFAS were not significant after FDR adjustment. When the overall PFAS exposure index (mean of standardized ln-PFAS concentrations) was considered, both baseline and annualized change measures were significantly associated with higher eGFR slopes (β = 0.402 and 0.331, respectively; *p* < 0.05). Potential non-linear dose–response relationships between PFAS exposure and annualized eGFR change were examined using polynomial regression terms. Significant quadratic or cubic terms were identified for a subset of compounds, indicating non-linear trends. The resulting dose–response patterns are depicted in Figure 2. For the linear fits (baseline linear PFOS, baseline branched PFOA, Δln-branched PFOS/Δt, baseline PFNA, baseline PFDA, and Δln-PFDA/Δt), the unadjusted scatterplots showed weakly negative trends, but the directions reversed after covariate adjustment, revealing positive associations consistent with the multivariable results in Figure 1. The quadratic fit for Δln-linear PFOA/Δt displayed an inverted-U pattern. The cubic fits (baseline linear PFOA, Δln-linear PFOS/Δt, and Δln-PFNA/Δt) exhibited gentle upward S-shaped curves.

**Figure 3:**
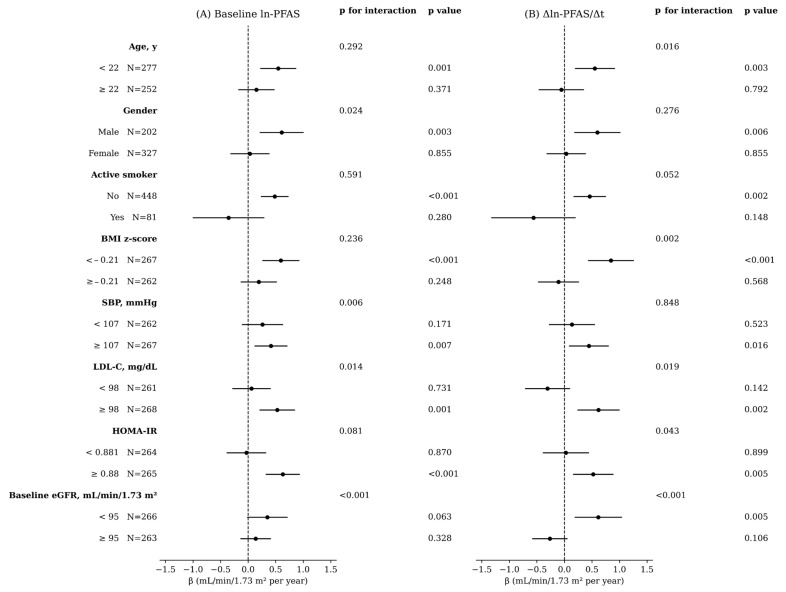
Annualized change model: ln-PFAS exposure index (baseline and Δln-PFAS exposure index/Δt) vs. annualized eGFR change (ΔeGFR/Δt; per 1-SD exposure) across different subgroups. (**A**) baseline ln-PFAS and (**B**) annualized change (Δln-PFAS/Δt). All models are adjusted for covariates defined in Model 1 (see text).

Figure 3 summarizes subgroup analyses of the associations between the ln-PFAS exposure index (both baseline and annualized change) and annualized eGFR change. Overall, similar subgroup patterns were observed for both baseline and Δln-PFAS/Δt measures, with stronger positive associations among younger participants (<22 years), males, non-smokers, and those with lower BMI z-scores, higher SBP, elevated LDL-C, higher HOMA-IR, and lower baseline eGFR. Despite these comparable trends, distinct patterns of statistical interaction were noted. For baseline PFAS exposure, significant effect modification was detected by gender, SBP, LDL-C, and baseline eGFR (all *p* for interaction < 0.05). For the annualized change in PFAS exposure index, significant interactions were observed for age, BMI z-score, LDL-C, HOMA-IR, and baseline eGFR. Detailed numerical estimates, including effect sizes and CI for individual PFAS, are provided in Supplementary Table S3.

**Figure 4:**
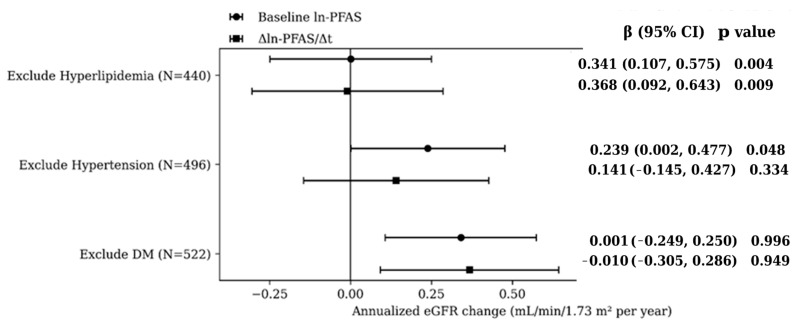
Sensitivity analyses: Annualized eGFR change per 1-SD increase in the standardized ln-PFAS exposure index after excluding selected comorbid conditions.

Figure 4 presents sensitivity analyses assessing the robustness of the associations between the PFAS exposure index and annualized eGFR change. The results generally supported the robustness of the main findings. Excluding participants with DM preserved significant positive associations for both baseline and longitudinal PFAS exposure, indicating that results were not driven by metabolic disease. In contrast, excluding individuals with hypertension and hyperlipidemia diminished associations.

Supplemental Figure S1 shows that higher baseline and increased PFAS exposure were generally associated with higher follow-up eGFR after multivariable adjustment. Significant positive associations were observed for linear PFOA, linear PFOS, branched PFOS, PFNA, and PFDA (q < 0.05). The overall PFAS exposure index also remained positively related to follow-up eGFR in both baseline and change models. Supplemental Table S4 summarizes the linear mixed-effects models for eGFR trajectories. Higher baseline PFAS concentrations, particularly linear PFOA, PFNA, N-MeFOSAA, and the PFAS exposure index, were significantly associated with higher eGFR intercepts (all *p* < 0.05). Most compounds showed no significant time interactions, indicating little evidence of slope modification. Only PFDoA and N-MeFOSAA exhibited significant negative interactions with time, suggesting a slightly faster eGFR decline among participants with higher baseline levels.

## 4. Discussion

In this longitudinal cohort of Taiwanese adolescents and young adults, we observed a decade-long decline in plasma concentrations of several legacy PFAS, reflecting the effectiveness of global and domestic regulatory actions [4,5,6]. Despite these overall reductions, higher baseline and increasing PFAS levels, particularly linear PFOA, linear and branched PFOS, PFNA, and PFDA, were consistently associated with greater increases in eGFR over time. The concurrent significance of both baseline and temporal change measures points to a sustained physiological response rather than transient exposure variability. Further, polynomial analyses revealed mild non-linear exposure–response relationships for several PFAS, indicating that the renal effects may not follow a strictly linear pattern across exposure ranges. Moreover, the consistency of results across multiple sensitivity analyses reinforces the robustness of these findings. Taken together, the positive associations observed in this young cohort may reflect an early stage of glomerular hyperfiltration, representing a compensatory response to renal stress. Overall, our findings highlight the need to adopt a life-course perspective when investigating PFAS-related renal effects.

Although overall PFAS concentrations declined at the population level during follow-up, this trend is distinct from the exposure–response associations examined in our regression analyses. Population-level declines reflect regulatory and environmental changes, whereas our models evaluate whether individuals with relatively higher PFAS exposure or greater longitudinal changes experienced different kidney function trajectories within the cohort. These processes may coexist, particularly in young populations with limited variability in kidney outcomes, and therefore diffuse scatterplots do not preclude the presence of adjusted associations. Our study applied complementary analytical frameworks to delineate PFAS–renal associations. The annualized change model (Figure 1) evaluated whether baseline PFAS and their temporal trajectories (Δln-PFAS/Δt) were related to the rate of kidney function change (ΔeGFR/Δt), capturing dynamic co-variation between exposure and renal response. In contrast, the follow-up eGFR model (Supplementary Figure S1) used cumulative PFAS change (Δln-PFAS) to predict the final filtration level after accounting for baseline eGFR and follow-up duration, thereby reflecting the association between cumulative PFAS change and the attained level of kidney function. These dual modeling approaches are consistent with prior methodological work demonstrating that slope-based models assess rates of decline, whereas level-based models evaluate end-of-study outcomes [39]. The results from both approaches were largely consistent, suggesting that PFAS exposure influences renal filtration both dynamically and cumulatively. The subsequent mixed-effects analysis (Supplementary Table S4) further extended this framework by incorporating repeated eGFR measures to capture within-person trajectories [40]. The lack of significant baseline PFAS × time interactions implies that baseline PFAS primarily affected filtration levels rather than altering the longitudinal rate of decline. Collectively, these findings raise the hypothesis that PFAS-related elevations in eGFR may reflect early alterations in renal hemodynamics, such as hyperfiltration or a compensatory response to renal stress. However, the clinical significance of this pattern cannot be determined from the present observational data.

In our cohort of Taiwanese adolescents and young adults, both baseline and increasing PFAS concentrations were positively associated with eGFR. Experimental and toxicological studies support several biological mechanisms that could underlie such PFAS-associated hyperfiltration. Long-chain PFAS such as PFOA and PFOS can activate PPARα, promoting fatty-acid β-oxidation and metabolic reprogramming in renal tissue, which may alter renal energy demand and hemodynamic balance, potentially contributing to early hyperfiltration [15,18]. PFAS also induce oxidative stress and impair antioxidant defense pathways such as nuclear factor erythroid 2–related factor 2, resulting in endothelial dysfunction and altered glomerular microvascular tone [16]. In addition, PFAS interact with renal organic anion transporters in proximal tubular cells, potentially altering proximal solute handling and reducing sodium chloride delivery to the macula densa, which could blunt tubuloglomerular feedback and transiently elevate glomerular filtration [17]. Furthermore, the high protein-binding affinity and lipophilicity of PFAS prolong systemic retention and sustain renal exposure [41]. Collectively, these processes suggest that PFAS-related hyperfiltration may represent an early adaptive renal response to chemical stress, which, if persistent, could evolve into glomerular injury and later functional decline.

An alternative explanation is potential bidirectionality between PFAS and kidney function. PFAS toxicokinetics are closely linked to renal handling. PFAS are primarily eliminated via the urine, and their circulating levels are influenced by glomerular filtration, together with active tubular transport and reabsorption [42]. Therefore, kidney function may act as a determinant of measured plasma PFAS concentrations, raising the possibility of reverse causation and GFR-related confounding in PFAS epidemiology [43]. Notably, higher eGFR would be expected to increase renal clearance and thereby lower plasma PFAS concentrations, which would tend to bias associations toward the null or an inverse direction rather than generate the positive associations observed here; nonetheless, we interpret our findings cautiously and emphasize the need for future studies that explicitly address bidirectionality.

The heterogeneous associations observed across individual PFAS compounds highlight the importance of considering chain length, isomeric structure, and functional chemistry when interpreting renal outcomes. In the Swedish PIVUS study, long-chain PFAS such as PFNA, PFDA, and PFUdA were positively associated with eGFR, whereas shorter-chain analogs or sulfonamide derivatives showed weaker or null associations [26]. This pattern aligns with toxicokinetic evidence that long-chain PFAS exhibit higher protein binding affinity and slower renal clearance, resulting in greater systemic persistence [41,44]. However, our findings did not fully follow this chain-length gradient. In our cohort, C8 compounds, linear PFOA, linear PFOS, and branched PFOS showed the most consistent positive associations with eGFR, whereas longer-chain PFUdA (C11) and PFDoA (C12) were not significant in either model. N-MeFOSAA, an eight-carbon sulfonamide precursor of PFOS, was also null despite a similar chain length. These discrepancies likely reflect differences in functional group chemistry, protein binding, and renal transporter affinity, as well as the limited variability of very long-chain PFAS due to their strong lipophilicity and slow plasma–tissue exchange [45]. Collectively, these findings suggest that PFAS–renal associations are compound-specific rather than monotonic across chain lengths. The divergence from the PIVUS findings may reflect differences in age distribution and exposure dynamics between cohorts. Older adults in PIVUS are more likely to experience age-related eGFR decline and may have a narrower exposure range, whereas our younger cohort may exhibit different renal hemodynamic patterns and larger within-person changes in PFAS over time. Together, these factors could contribute to the more consistently positive associations observed in our analyses. Extending this comparison to other longitudinal studies further illustrates how PFAS–renal associations vary across exposure levels and life stages. In the Northeastern Taiwan Community Medicine Research Cohort (adult population, four-year follow-up), branched PFOS and PFHxS predicted greater eGFR decline over time, whereas some long-chain PFAS, such as PFNA, showed negative associations only at baseline [25]. Similarly, the Fernald Community Cohort in the United States (middle-aged adults with moderate-to-high PFAS exposure) found inverse longitudinal associations for PFNA, PFHxS, and PFDeA with eGFR [24]. Together, these contrasts may reflect differences in age, exposure intensity, baseline kidney function, and renal reserve. In this context, the eGFR elevations observed in our younger cohort may be consistent with early renal hemodynamic alterations (e.g., hyperfiltration) or compensatory stress responses, whereas older or more highly exposed populations may show patterns more consistent with subsequent functional decline.

Our results showed that linear and branched PFOS showed similar associations with kidney function. Experimental evidence indicates that both sulfonate and carboxylate PFAS can interact with renal organic anion transporters in proximal tubular cells, supporting a shared mechanism for tubular reabsorption and secretion that may contribute to their renal accumulation and persistence [17]. However, linear and branched PFOA showed divergent associations in our study. The much lower concentrations of branched PFOA, with many values near the detection limit, likely reduced statistical power and increased exposure misclassification. These measurement constraints may explain its weaker or null association with kidney function rather than a true biological difference between isomers.

The mild curvature observed for selected PFAS (quadratic/cubic patterns) is biologically plausible within a low-dose toxicology framework, where non-monotonic dose–response relationships have been described for endocrine-active chemicals [46]. For PFAS, several processes could contribute to non-linearity, including receptor-mediated signaling with feedback regulation, concentration-dependent binding to serum proteins, and transporter-mediated renal handling that may vary across exposure ranges [47]. In addition, concentration-dependent associations have been reported in PFAS-related endocrine outcomes, supporting the plausibility that effect estimates may differ across exposure strata rather than follow a single monotonic slope [48].

Subgroup analyses revealed that the positive associations between PFAS exposure and annualized eGFR change were not uniform across the population. Stronger relationships were observed among participants who were younger, male, and those with lower BMI z-scores or baseline eGFR, as well as individuals with higher SBP, LDL-C, and HOMA-IR. Younger participants may have greater renal reserve and compensatory capacity, consistent with evidence that glomerular hyperfiltration is more common in healthy young adults and may represent an adaptive hemodynamic response to metabolic or chemical stress [49]. Similarly, males and individuals with higher cardiometabolic indicators may experience enhanced glomerular perfusion and filtration pressure, amplifying PFAS-associated increases in eGFR [50,51]. In contrast, participants with higher BMI or higher baseline eGFR showed weaker or null associations, consistent with a ceiling effect in filtration capacity. Notably, subgroup-specific *p*-values and *p* for interaction were not always concordant. Subgroup *p*-values assess associations within each stratum, whereas the *p* for interaction evaluates whether effect sizes differ significantly across strata. Differences between these tests are expected because they address distinct hypotheses and have unequal statistical power [52]. For example, younger participants showed significant positive β estimates for both baseline and Δln-PFAS/Δt, but only the latter yielded a significant interaction, suggesting stronger renal effects of exposure changes during youth. Males exhibited stronger baseline associations without significant interactions, implying similar effect directions but different magnitudes. In contrast, LDL-C and baseline eGFR showed consistent significance across both tests, confirming their role as robust modifiers of PFAS–eGFR relationships. Collectively, the subgroup estimates and corresponding interaction tests indicate that PFAS-related hyperfiltration is shaped by age, sex, metabolic activity, and baseline renal function. Although we conducted extensive subgroup analyses, these findings should be interpreted as exploratory. Statistical significance—particularly interaction *p*-values—depends on sample size and variability and does not necessarily imply biological importance. These results should be viewed as hypothesis-generating and warrant confirmation in larger studies with prespecified effect-modification hypotheses.

From an environmental risk-assessment perspective, the observed PFAS-associated elevation in eGFR in young people should be interpreted as a potential early functional perturbation under chronic, low-level exposure rather than a beneficial adaptation. While its clinical significance cannot be established in this study, these findings support continued PFAS biomonitoring in younger populations and upstream exposure reduction (e.g., tighter control of industrial releases and accelerated substitution with safer alternatives). From a clinical perspective, regular surveillance of kidney function and metabolic parameters (such as eGFR, blood pressure, and lipid profiles) may help identify individuals at higher susceptibility [16]. At the policy level, these results reinforce the importance of evaluating and phasing out persistent PFAS in high-tech manufacturing while promoting safer fluorine-free alternatives, consistent with national and global regulatory roadmaps that call for lifecycle controls and remediation of PFAS [53].

This study possesses several important strengths. First, it utilized a decade-long longitudinal design within a well-characterized cohort of adolescents and young adults, enabling the evaluation of long-term changes in plasma PFAS concentrations and kidney function during a physiologically dynamic life stage. Second, repeated measurements of PFAS levels provided a unique opportunity to assess intra-individual exposure trajectories, which are rarely available in younger populations. Third, the inclusion of both linear and branched PFAS isomers, analyzed under strict laboratory quality control, strengthened the validity and chemical specificity of exposure assessment. Fourth, the study incorporated detailed cardiometabolic profiling, allowing for comprehensive covariate adjustment and subgroup analyses that revealed potential effect modification by metabolic factors. Finally, extensive sensitivity analyses—including alternative model specifications, exclusion of participants with chronic conditions, and use of different exposure metrics—demonstrated the robustness and reproducibility of the findings.

Nonetheless, several limitations should be acknowledged. First, the cohort’s relatively young age and low prevalence of CKD or metabolic disease limited variability in kidney outcomes, potentially attenuating adverse associations. Second, PFAS concentrations were assessed at only two time points, restricting the ability to capture short-term fluctuations or non-linear exposure patterns. Third, although the z-score-based composite PFAS index provides a parsimonious summary of overall mixture burden, it assumes equal weighting across constituents and may mask compound-specific heterogeneity, including differential or opposing associations across PFAS. Therefore, the composite index should be interpreted as a mixture-level summary rather than as a substitute for compound-specific inference; accordingly, we report individual PFAS results alongside the index to aid interpretation. Fourth, although extensive covariates were included, residual confounding from unmeasured factors such as dietary habits, co-exposures to other environmental pollutants, or genetic predispositions cannot be excluded. Finally, loss to follow-up and the metropolitan sampling frame may limit generalizability to populations with different environmental or socioeconomic contexts.

## 5. Conclusions

In this prospective cohort of Taiwanese adolescents and young adults, plasma concentrations of several legacy PFAS declined over a decade, reflecting global regulatory impacts. Despite these reductions, higher baseline and increasing PFAS concentrations, particularly linear PFOA, linear and branched PFOS, PFNA, and PFDA, were associated with greater increases in eGFR over time. Additional analyses also suggested mild non-linear exposure–response relationships, consistent with quadratic and cubic trends observed for selected PFAS. The concordant associations observed for both baseline levels and exposure changes may reflect early renal hemodynamic alterations (e.g., hyperfiltration) or compensatory stress responses during young adulthood; however, the clinical significance of elevated eGFR cannot be determined from the present observational data. Associations appeared more pronounced among individuals with greater metabolic and physiological vulnerability. Overall, our findings indicate that both long-term PFAS burdens and exposure dynamics influence kidney function in early life. Continued follow-up is warranted to clarify whether early elevations in eGFR precede later decline and to inform exposure mitigation strategies across developmental stages.

## Figures and Tables

**Table 1:** Baseline characteristics of participants in this study (*N* = 529).

	2006–2008		2017–2019	
Variables	Mean/Numbers	SD/%	Mean/Numbers	SD/%
Age (year)	21.14	3.58	31.29	3.48
Female	327	61.8	327	61.8
Exercise habit	162	30.6	284	53.7
Active smoker	64	12.1	80	15.1
Medical history:				
DM	7	1.3	17	3.2
Hypertension	33	6.2	34	6.4
Hyperlipidemia	89	16.4	158	29.9
CKD	7	1.3	6	1.1
BMI (kg/m^2^)	21.70	3.93	22.74	4.37
SBP (mmHg)	106.54	13.77	115.13	13.84
LDL-C (mg/dL)	100.95	30.61	113.49	31.65
HOMA-IR	1.21	1.65	2.31	2.05
eGFR (mL/min/1.73 m^2^)	95.51	15.58	109.55	15.75

**Table 2:** Longitudinal trends of PFAS levels in the YOTA Cohort.

	2006–2008 (*N* = 529)	2017–2019 (*N* = 529)	Percent Change (%)	*p* Value
PFAS (ng/mL)	Geometric Mean (SD)	Geometric Mean (SD)		
Linear PFOA	2.31 (1.68)	1.82 (1.71)	−21.2	<0.001
Branched PFOA	0.06 (1.64)	0.07 (1.71)	16.7	0.017
Linear PFOS	3.88 (2.21)	3.82 (2.09)	−1.5	0.669
Branched PFOS	0.16 (2.08)	0.07 (2.33)	−56.3	<0.001
PFNA	0.99 (1.54)	0.71 (1.55)	−28.3	<0.001
PFHxS	0.68 (1.93)	0.71 (1.74)	4.4	0.144
PFDA	0.80 (2.02)	0.49 (1.68)	−38.8	<0.001
PFUdA	0.71 (1.79)	0.39 (1.84)	−45.1	<0.001
PFDoA	0.12 (1.80)	0.09 (2.00)	−25	<0.001
N-MeFOSAA	0.30 (2.71)	0.06 (4.20)	−80	<0.001
PFHpA	0.06 (2.23)	0.05 (2.56)	−16.7	0.001

## Data Availability

The data presented in this study are available on request from the corresponding author due to ethical and data protection restrictions, as the underlying dataset contains de-identified individual-level information derived from human participants and may require appropriate institutional approvals prior to sharing.

## Supplementary Materials

The following supporting information can be downloaded at: https://www.mdpi.com/article/10.3390/jox16010016/s1, Table S1: Linearity and sensitivity of PFAS by LC–MS/MS; Table S2: Accuracy and precision of the on-line SPE LC-MS/MS method for quantifying PFAS in the pooled plasma; Table S3: Annualized change model: ln-PFAS exposure index (baseline and Δln- PFAS exposure index/Δt) vs. annualized eGFR change (ΔeGFR/Δt; per 1-SD exposure) across different subgroups; Table S4: Linear mixed-effects models of baseline ln-PFAS and its interaction with time for eGFR trajectory (exposures standardized to 1-SD; time in years, baseline set to 0); Figure S1: Follow-up eGFR per 1-SD increase in standardized ln-PFAS. Panels show β estimates and 95% CI from multivariable linear models for (A) baseline ln-PFAS and (B) PFAS change (Δln-PFAS). Nominal *p*-values and FDR-adjusted q-values are shown to the right. All models are adjusted for covariates defined in Model 1 (see text) and follow-up duration. The PFAS exposure index is the average of standardized (z-score transformed) PFAS concentrations. References [54,55,56] are cited in the Supplementary Materials.
