# Supplementary material for: Long-Term Per- and Polyfluoroalkyl Substances Exposure and Kidney Function in Taiwanese Adolescents and Young Adults: A 10-Year Prospective Cohort Study
Source: J Xenobiot. 2026 Jan 21;16(1):16. doi: 10.3390/jox16010016 (PMC12922100; doi:10.3390/jox16010016)
Supplement: Supplementary file 1 [file jox-16-00016-s001.zip › jox-4087395-supplementary.pdf]

Journal of *Xenobiotics*

MDPI

Supplementary Materials: Long-Term Per- and Polyfluoroalkyl Substances Exposure and Kidney Function in Taiwanese Adolescents and Young Adults: A 10-Year Prospective Cohort Study

Chien-Yu Lin, Hui-Ling Lee, and Ta-Chen Su

1.1. Study population and data collection

Between 1992 and 2000, a substantial number of school-age children in grades 1 to 12 in Taiwan, underwent annual urine screenings conducted by the Chinese Foundation of Health. The screenings involved the use of urine strips to detect abnormalities related to proteinuria, glycosuria, or hematuria. Students who displayed abnormal results in at least two of these screenings were subjected to a third urine test and a general health check-up. Among the extensive pool of screened children, a total of 103,756 students received these health check-ups and the third urine screening. This group was further categorized into 9,227 students with elevated blood pressure and 94,529 students with normal blood pressure [1]. Subsequently, from 2006 to 2008, we initiated the YOung TAiwanese Cohort (YOTA) study. This cohort was formed by selecting students from the earlier 1992-2000 urine screening population. During the follow-up phase, invitation letters were sent to eligible students in the Taipei area. For individuals with childhood elevated blood pressure, telephone interviews were conducted to invite them for a follow-up health examination. In contrast, no telephone interviews were conducted with normotensive students. Among the group of 707 subjects with elevated blood pressure in childhood, 303 individuals completed the follow-up health examinations, yielding a response rate of 42.9%. In the normotensive group of 6,390 subjects from childhood, 486 individuals completed the follow-up health examinations, resulting in a response rate of 7.6%. Additionally, to assess the impact of environmental factors on the age of exposure, 97 subjects were recruited as "best friend controls" during the cohort follow-up period. In total, the study included 886 subjects and all participants had baseline serum PFAS level data. [2]. Between 2017 and 2019, 542

YOTA participants were recruited to examine how living environments and dietary habits affect cardiometabolic disorders [3]. Among these 542 participants, 11 were excluded due to unavailable serum samples for PFAS measurements. All subjects provided written informed consent, and the research projects were approved by the Research Ethics Committee of the National Taiwan University (IRB No: 9561705054 and 201604089RINA). We verified that all methods were conducted in accordance with the appropriate regulations and guidelines.

1.2. Measurement of PFAS concentrations:

The plasma samples collected at baseline (2006-2008) and during follow-up (2017-2019) were stored at -80°C before undergoing analysis. These specimens are tested in the Laboratory of the Department of Chemistry at Fu Jen Catholic University.

Chemicals

Perfluorohexane sulfonic acid (L-PFHxS), Perfluorooctane sulfonic acid (L-PFOS), Perfluoroheptanoic acid (PFHpA), Perfluorooctanoic acid (PFOA), Perfluorononanoic acid (PFNA), Perfluorodecanoic acid (PFDA), Perfluoroundecanoic acid (PFUdA), Perfluorododecanoic acid (PFDoA), N-methylperfluorooctane sulfonamide acetic acid (N-MeFOSAA), Sodium perfluoro-1-methylheptanesulfonate (1m-PFOS),Sodium perfluoro-5-methylheptanesulfonate (5m-PFOS), Sodium perfluoro-6-methylheptanesulfonate (6m-PFOS), Sodium perfluoro-3,5-dimethylhexanesulfonate (3,5m2-PFOS), Sodium perfluoro-4,5-dimethylhexanesulfonate (4,5m2-PFOS), Sodium perfluoro-4,4-dimethylhexanesulfonate (4,4m2-PFOS), Sodium perfluoro-5,5-dimethylhexanesulfonate (5,5m2-PFOS), Perfluoro-5-methylheptanoic acid (5m-PFOA), Perfluoro-6-methylheptanoic acid (6m-PFOA), Perfluoro-3,5-dimethylhexanoic acid (3,5m2-PFOA), Perfluoro-4,5-dimethylhexanoic acid (4,5m2-PFOA), Perfluoro-4,4-dimethylhexanoic acid (4,4m2-PFOA), Perfluoro-5,5-dimethylhexanoic acid (5,5m2-PFOA), Perfluoro-n-[1,2,3,4-$^{13}C_4$] heptanoic acid ($^{13}C_4$-M4PFHpA), Sodium perfluoro-1-hexane[$^{18}O_2$]sulfonate ($^{18}O_2$-MPFHxS), Sodium

perfluoro-1-[$^{13}C_8$]octanesulfonate ($^{13}C_8$-M8PFOS), Perfluoro-n-[$^{13}C_8$]octanoic acid ($^{13}C_8$-M8PFOA), Perfluoro-n-[1,2,3,4,5-$^{13}C_5$]nonanoic acid ($^{13}C_5$-MPFNA), Perfluoro-n-[1,2-$^{13}C_2$]undecanoic acid ($^{13}C_2$-MPFUdA), Perfluoro-n-[1,2-$^{13}C_2$]dodecanoic acid ($^{13}C_2$- MPFDoA), N-methyl-d_3-perfluoro-1-octanesulfonamidoacetic acid (d_3-N-MePFOSAA) were obtained from Wellington laboratories (Guelph, ON, Canada). Gradient-grade acetonitrile (ACN) and formic acid (FA) were purchased from Sigma-Aldrich (St. Louis, Missouri, USA), and all aqueous solutions were prepared using deionized water (18.2 MΩ•cm) filtered through a Millipore Milli-Q water system. In the current study, the level of branched PFOS (B-PFOS) was the sum of 3,5m_2-PFOS, 4,5m_2-PFOS, 5,5m_2-PFOS, and 4,4m_2-PFOS (1m-PFOS, 5m-PFOS, and 6m-PFOS were not detectable) while the level of branched PFOA (B-PFOA) was the sum of 5m-PFOA, 6m-PFOA, 4,4m_2-PFOA, and 5,5m_2-PFOA (3,5m_2-PFOA and 4,5m_2-PFOA were not detectable).

Sample preparation

Fifty microliters of human plasma were added to 100 μL acetonitrile to remove protein before centrifuging at 12,000 rpm at 4°C for 10 min. The supernatant was diluted and spiked with a fixed amount of 1.0 ng/mL $^{18}O_2$-PFHxS, $^{13}C_4$-PFHpA, $^{13}C_8$-PFOA, $^{13}C_8$-PFOS, $^{13}C_5$-PFNA, $^{13}C_2$-PFUdA, $^{13}C_2$-PFDoA and d_3-N-MeFOSAA as internal standards, which were used as internal standards, and transferred into sample vials for analysis.

Automated on-line SPE LC-MS/MS system

The analytical method presented here consisted of two HPLC pumps to achieve the on-line SPE LC-MS/MS system. The automated column-switching system included a binary pump (Agilent 1260; Agilent Technology, U.S.A.), an extraction column (Inertsil ODS-3 33 × 4.6 mm, 5 μm), and a two-position microelectric valve actuator (Valco Instrument Co., Ltd.). The gradient system for extraction consisted of mobile phase A (1% MeOH, v/v with 0.1% FA) and mobile phase B (90% MeOH, v/v with 0.1% FA) at a flow rate of 0.5 mL/min. The valve actuator was controlled by the Analyst 1.6.2™ software (Applied Biosystems, MDS SCIEX, Concord, Ontario, Canada). Separation was performed by an additional quaternary pump (Agilent 1260; Agilent Technology) and analytical column (Agilent Eclipse Plus C18,

100 × 4.6 mm, 3.5 μm). The gradient system consisted of mobile phase A (10 mM ammonium formate pH 3.5) and mobile phase B (100% MeOH) at a flow rate of 0.35 mL/min. The injection volume was 40 μL. The detailed information for the on-line SPE LC-MS/MS system is summarized in elsewhere.

The analytical column was directly connected to an API 3000 (Applied Biosystems, MDS SCIEX, Concord, Ontario, Canada). Mass spectrometry detection was conducted in negative electrospray ionization mode at −4000 V for ion spray voltage. The optimal ion source parameters were nebulizer gas at 13 psi and curtain gas at 10 psi, collision-assisted dissociation at 8 psi, and source heater probe temperature at 500°C. The fragmentations of each precursor for multiple reaction monitoring (MRM) scan mode. The linearity of the method was determined by the calibration curves constructed for each analyte, and was based on the lower limits of quantification and calibration results. Calibration curves were calculated by plotting a peak-area ratio of the standards and IS against the concentration, according to the following formula: Ratio = (ASTD in neat solvent) / (AIS in neat solvent) using least-squares linear regression. The LOD was defined as a S/N ratio of 3, which was found in 0.002 and 0.150 ng/mL. The LOQ was defined as a S/N ratio of 10, which was found in 0.006 and 0.500 ng/ mL. The results are summarized in Table S1.

The precision and accuracy, to variability of intra- and inter-day measurements was assessed via the recoveries for PFAS by analyzing replicates (n = 5) spiked with three different known levels into pooled plasma, and by calculating the CV (coefficient of variation). The accuracy was expressed as recovery (%) = [(measured concentration – blank plasma matrix concentration)/spiked concentration] × 100%. The detection accuracy (n = 5) of the PFAS ranged from 99.34-117.52% and 98.30-123.74%, the precision ranged from 0.32-10.40% and 0.65- 15.32%, respectively. The results are summarized in Table S2.

The detection rates varied among specific PFAS compounds in two different sample periods. In the samples collected between 2006 and 2008, all linear and branched forms of PFOA and PFOS, PFHxS, PFNA, PFDA, PFUdA, and PFDoA showed a 100% detection rate. Additionally, detection rates remained relatively high for N-MeFOSAA and

PFHPA, ranging from 92.3% to 99.2%. For the samples collected between 2017 and 2019, all linear and branched forms of PFOA and PFOS, PFHxS, PFNA, PFDA, PFUdA, PFDoA, and N-MeFOSAA had a 100% detection rate, while PFHPA exhibited a slightly lower but still substantial detection rate of 89.9%. Analytic results falling below the lower limit of detection were imputed using a value equivalent to the limit of detection divided by the square root of 2. Importantly, all laboratory analyses were conducted impartially by investigators unaware of the characteristics of the study subjects.

Table S1. Linearity and sensitivity of PFAS by LC–MS/MS.

Analyte	Calibration range (ng/mL)	Calibration curves	Correlation coefficient (r^2)	LOD [a] (ng/mL)	LOQ [b] (ng/mL)
N-MeFOSAA	0.05-5.0	y = 0.4030x + 0.0104	0.9987	0.010	0.032
PFHpA	0.05-5.0	y = 1.0824x – 0.0050	0.9993	0.011	0.037
PFOA	0.05-5.0	y = 0.9462x – 0.0056	0.9986	0.005	0.016
PFNA	0.05-5.0	y = 1.0478x + 0.0001	0.9996	0.016	0.053
PFDA	0.05-5.0	y = 0.7661x – 0.0082	0.9995	0.002	0.006
PFUdA	0.05-5.0	y = 1.2469x – 0.0417	0.9992	0.020	0.066
PFDoA	0.05-5.0	y = 1.0628x + 0.0005	0.9991	0.004	0.014
PFHxS	0.05-5.0	y = 0.3787x – 0.0084	0.9988	0.026	0.086
PFOS	0.05-5.0	y = 2.7109x – 0.0644	0.9984	0.017	0.057
5m-PFOA	0.05-5.0	y = 2.1404x – 0.0041	0.9988	0.003	0.011
6m-PFOA	0.05-5.0	y = 1.7027x + 0.0081	0.9996	0.004	0.013
4,4m$_2$-PFOA	0.05-5.0	y = 1.9866x + 0.0292	0.9998	0.007	0.022
5,5m$_2$-PFOA	0.05-5.0	y = 2.0665x + 0.0105	0.9995	0.004	0.014
4,5m$_2$-PFOA	0.05-5.0	y = 0.4328x + 0.0167	0.9998	0.074	0.248
4,5m$_2$-PFOA	0.05-5.0	y = 1.0167x + 0.0306	0.9998	0.016	0.052
3,5m$_2$-PFOA	0.05-5.0	y = 0.3714x + 0.0144	0.9996	0.046	0.152
3,5m$_2$-PFOA	0.05-5.0	y = 0.2268x + 0.0158	0.9987	0.011	0.038
1m-PFOS	0.05-5.0	y = 1.1896x – 0.0785	0.9977	0.007	0.022
5,5m$_2$-PFOS	0.05-5.0	y = 0.3032x – 0.0209	0.9986	0.060	0.200
1m/5m/6m-PFOS	0.05-5.0	y = 12.0394x – 0.2340	0.9998	0.013	0.042
1m/5m/6m-PFOS	0.05-5.0	y = 7.2368x – 0.2003	0.9997	0.010	0.032
1m/5m/6m-PFOS	0.05-5.0	y = 0.1362x – 0.0098	0.9964	0.150	0.500
3,5m$_2$/4,5m$_2$/4,4m$_2$/5,5m$_2$-PFOS	0.05-5.0	y = 3.9068x – 0.0607	0.9916	0.006	0.021
3,5m$_2$/4,5m$_2$/4,4m$_2$/5,5m$_2$-PFOS	0.05-5.0	y = 1.4119x – 0.0103	0.9986	0.008	0.028
3,5m$_2$/4,5m$_2$-PFOS	0.05-5.0	y = 0.1621x – 0.0072	0.9991	0.023	0.077

[a]LOD: Limit of detection, [b]LOQ: Limit of quantification

Table S2. Accuracy and precision of the on-line SPE LC-MS/MS method for quantifying PFAS in the pooled plasma.

Analyte	Spiked (ng/mL)	Found ± SD [b] (ng/mL)	Intra-day (n = 5)		Inter-day (n = 15)	
			Recovery [a] (%)	CV [c] (%)	Recovery [a] (%)	CV [c] (%)
PFHxS	0.0	0.55 ± 0.02				
	0.2	0.75 ± 0.00	101.10	0.77	102.04	1.28
	0.6	1.16 ± 0.01	102.35	1.22	102.90	1.96
	1.2	1.76 ± 0.01	101.21	0.88	102.35	1.35
PFOS	0.0	6.55 ± 0.11				
	2.0	8.60 ± 0.02	102.69	1.06	102.35	1.11
	4.0	10.61 ± 0.02	101.53	0.60	101.96	1.44
	6.0	12.60 ± 0.03	100.76	0.41	100.66	1.49
PFHpA	0.0	0.00 ± 0.00				
	0.2	0.20 ± 0.00	102.28	0.88	102.61	1.17
	0.6	0.61 ± 0.01	101.92	0.92	103.35	2.06
	1.2	1.22 ± 0.01	101.55	0.76	100.96	1.75

PFOA	0.0	1.82 ± 0.11				
	0.2	1.99 ± 0.00	102.48	1.32	102.70	1.56
	0.6	2.40 ± 0.01	102.51	1.07	103.19	1.16
	1.2	3.00 ± 0.01	101.57	0.82	102.29	1.68
PFNA	0.0	0.83 ± 0.10				
	0.2	1.06 ± 0.00	101.29	1.34	103.90	2.20
	0.6	1.46 ± 0.01	104.90	1.25	106.97	3.76
	1.2	2.07 ± 0.02	103.36	1.62	102.33	1.61
PFDA	0.0	0.50 ± 0.05				
	0.2	0.80 ± 0.01	107.32	3.40	112.89	6.44
	0.6	1.16 ± 0.02	111.70	3.24	108.25	4.12
	1.2	2.12 ± 0.06	107.38	4.54	115.51	6.47
PFUdA	0.0	0.45 ± 0.03				
	0.2	0.66 ± 0.00	103.19	1.57	101.85	2.03
	0.6	1.07 ± 0.01	102.94	1.85	100.78	2.16
	1.2	1.69 ± 0.02	102.69	1.28	100.32	2.44
PFDoA	0.0	0.07 ± 0.01				
	0.2	0.27 ± 0.00	100.89	0.32	100.99	0.65
	0.6	0.67 ± 0.01	100.84	1.00	100.42	1.07
	1.2	1.28 ± 0.01	100.80	0.41	101.23	0.86
N-MeFOSAA	0.0	0.13 ± 0.00				
	0.2	0.34 ± 0.01	101.11	2.49	102.75	2.13
	0.6	0.74 ± 0.01	100.40	0.96	101.87	2.24
	1.2	1.35 ± 0.01	101.31	1.19	102.33	2.28
6m-PFOA	0.0	0.00 ± 0.00				
	0.2	0.21 ± 0.01	103.44	3.91	103.54	2.88
	0.6	0.63 ± 0.01	104.67	2.21	106.31	2.57
	1.2	1.20 ± 0.02	100.01	1.74	103.07	2.83
5,5m$_2$-PFOA	0.0	0.03 ± 0.00				
	0.2	0.43 ± 0.00	101.52	0.77	107.11	4.09
	0.6	1.29 ± 0.03	107.55	2.46	110.07	3.55
	1.2	2.58 ± 0.07	108.74	2.95	109.08	3.73
5m-PFOA	0.0	0.00 ± 0.00				
	0.2	0.21 ± 0.00	105.53	1.56	105.73	2.23
	0.6	0.63 ± 0.02	105.58	3.39	108.46	3.31
	1.2	1.29 ± 0.05	107.17	3.97	105.58	3.01
4,4m$_2$-PFOA	0.0	0.00 ± 0.00				
	0.2	0.20 ± 0.00	100.53	2.46	102.82	3.27
	0.6	0.63 ± 0.01	104.76	2.21	104.20	2.78
	1.2	1.21 ± 0.03	101.06	2.40	103.54	3.40
1m/5m/6m-PFOS	0.0	0.16 ± 0.00				
	0.2	0.37 ± 0.00	102.69	2.24	103.42	2.96
	0.6	0.78 ± 0.00	102.77	0.79	102.21	2.44
	1.2	1.37 ± 0.02	100.94	1.55	100.96	1.06
1m/5,5m$_2$-PFOS	0.0	0.24 ± 0.02				
	0.2	0.47 ± 0.02	106.42	4.42	104.21	5.08
	0.6	0.83 ± 0.03	100.46	1.27	100.80	15.32
	1.2	1.48 ± 0.08	103.86	4.15	109.90	10.78
ΣPFOS [d]	0.0	0.02 ± 0.00				
	0.2	0.22 ± 0.02	99.34	8.23	109.80	13.48
	0.6	0.62 ± 0.06	100.26	10.40	108.59	14.60
	1.2	1.34 ± 0.08	109.93	5.83	123.74	9.10
3,5m$_2$/4,5m$_2$-PFOS	0.0	0.00 ± 0.00				

	0.2	0.23 ± 0.02	112.72	7.22	117.21	9.99
	0.6	0.66 ± 0.02	110.69	3.03	98.30	14.38
	1.2	1.41 ± 0.15	117.52	10.37	110.80	10.68

[a] Recovery (%) = [(measured concentration – blank plasma matrix concentration)/spiked concentration] × 100%., [b] SD：Standard deviation, [c] CV：Coefficient of variation

[d] ΣPFOS：3,5m_2/4,5m_2/5,5m_2/4,4m_2-PFOS

Table S3. Annualized change model: ln- PFAS exposure index (baseline and Δln- PFAS exposure index /Δt) vs. annualized eGFR change (ΔeGFR/Δt; per 1-SD exposure) across different subgroups.

		**Annualized eGFR change (mL/min/1.73 m² per year)**					
		Baseline ln-PFAS			Δln- PFAS exposure index /Δt		
	N	β(95% CI)	P-value	P for int.	β(95% CI)	P-value	P for int.
Age, y				0.292			0.016
< 22	277	0.546 (0.220, 0.872)	0.001		0.552 (0.189, 0.916)	0.003	
≥ 22	252	0.150 (-0.180, 0.479)	0.371		-0.055 (-0.466, 0.356)	0.792	
Gender				0.024			0.276
Male	202	0.610 (0.211, 1.009)	0.003		0.598 (0.177, 1.019)	0.006	
Female	327	0.033 (-0.323, 0.390)	0.855		0.033 (-0.323, 0.390)	0.855	
Active smoker				0.591			0.052
No	448	0.483 (0.232, 0.733)	<0.001		0.458 (0.165, 0.752)	0.002	
Yes	81	-0.355 (-1.005, 0.295)	0.280		-0.561 (-1.327, 0.204)	0.148	
BMI z-score				0.236			0.002
< -0.21	267	0.593 (0.257, 0.929)	<0.001		0.844 (0.429, 1.259)	<0.001	
≥ -0.21	262	0.193 (-0.136, 0.522)	0.248		-0.108 (-0.481, 0.265)	0.568	
SBP, mmHg				0.006			0.848
< 107	262	0.261 (-0.113, 0.635)	0.171		0.136 (-0.282, 0.554)	0.523	
≥ 107	267	0.414 (0.115, 0.713)	0.007		0.444 (0.083, 0.805)	0.016	
LDL-C, mg/dL				0.014			0.019
< 98	261	0.061 (-0.288, 0.410)	0.731		-0.306 (-0.714, 0.103)	0.142	
≥ 98	268	0.529 (0.206, 0.852)	0001		0.619 (0.237, 1.002)	0002	
HOMA-IR				0.081			0.043
< 0.881	264	-0.030 (-0.389, 0.329)	0.870		0.027 (-0.391, 0.446)	0.899	
≥ 0.88	265	0.629 (0.319, 0.939)	<0.001		0.523 (0.156, 0.889)	0.005	
Baseline eGFR, mL/min/1.73 m²				<0.001			<0.001
< 95	266	0.349 (-0.019, 0.716)	0.063		0.615 (0.186, 1.045)	0.005	
≥ 95	263	0.137 (-0.139, 0.413)	0.328		-0.263 (-0.583, 0.057)	0.106	

Models adjust for age, sex, income, exercise, smoking, BMI z-score, SBP, LDL-C, HOMA-IR, and baseline eGFR.

*PFAS exposure index: the average of standardized (z-score transformed) concentrations of PFAS

Table S4. Linear mixed-effects models of baseline ln-PFAS and its interaction with time for eGFR trajectory (exposures standardized to 1-SD; time in years, baseline set to 0)

PFAS (n=529)	Main effect (β), 95% CI	P-value	Interaction effect (β), 95% CI	P-value
Linear PFOA	2.553 (1.204, 3.902)	<0.001	-0.020 (-0.158, 0.117)	0.771
Branched PFOA	-0.196 (-1.519, 1.127)	0.771	0.008 (-0.135, 0.151)	0.914
Linear PFOS	0.575 (-0.762, 1.913)	0.399	-0.081 (-0.219, 0.058)	0.252
Branched PFOS	0.918 (-0.463, 2.299)	0.779	-0.020 (-0.159, 0.119)	0.192
PFNA	2.410 (1.061, 3.760)	<0.001	-0.078 (-0.216, 0.060)	0.267
PFHxS	1.028 (-0.353, 2.409)	0.144	0.016 (-0.118, 0.151)	0.814
PFDA	1.122 (-0.200, 2.444)	0.096	0.029 (-0.110, 0.168)	0.680
PFUdA	1.000 (-0.318, 2.318)	0.137	0.018 (-0.121, 0.157)	0.797
PFDoA	1.230 (-0.094, 2.554)	0.069	-0.171 (-0.313, -0.029)	0.018
N-MeFOSAA	1.458(0.144, 2.773)	0.030	-0.174 (-0.318, -0.031)	0.017
PFHpA	-0.720 (-2.074, 0.634)	0.297	0.073 (-0.067, 0.213)	0.307
PFAS exposure index*	3.420 (0.967, 5.873)	0.006	-0.113 (-0.366, 0.140)	0.382

Models adjusted for Model 1 except baseline eGFR. Fixed effects include time (years since baseline; baseline coded 0), baseline standardized ln-PFAS (per 1-SD), and their interaction (time × ln-PFAS); random intercept by participant; estimated by REML. (No baseline eGFR term is included in LMM because eGFR at baseline is part of the outcome.)

Figure S1. Follow-up eGFR per 1-SD increase in standardized ln-PFAS. Panels show β estimates and 95% CI from multivariable linear models for (A) baseline ln-PFAS and (B) PFAS change (Δln-PFAS). Nominal p-values and FDR-adjusted q-values are shown to the right. All models are adjusted for covariates defined in Model 1 (see text) and follow-up duration. The PFAS exposure index is the average of standardized (z-score transformed) PFAS concentrations.

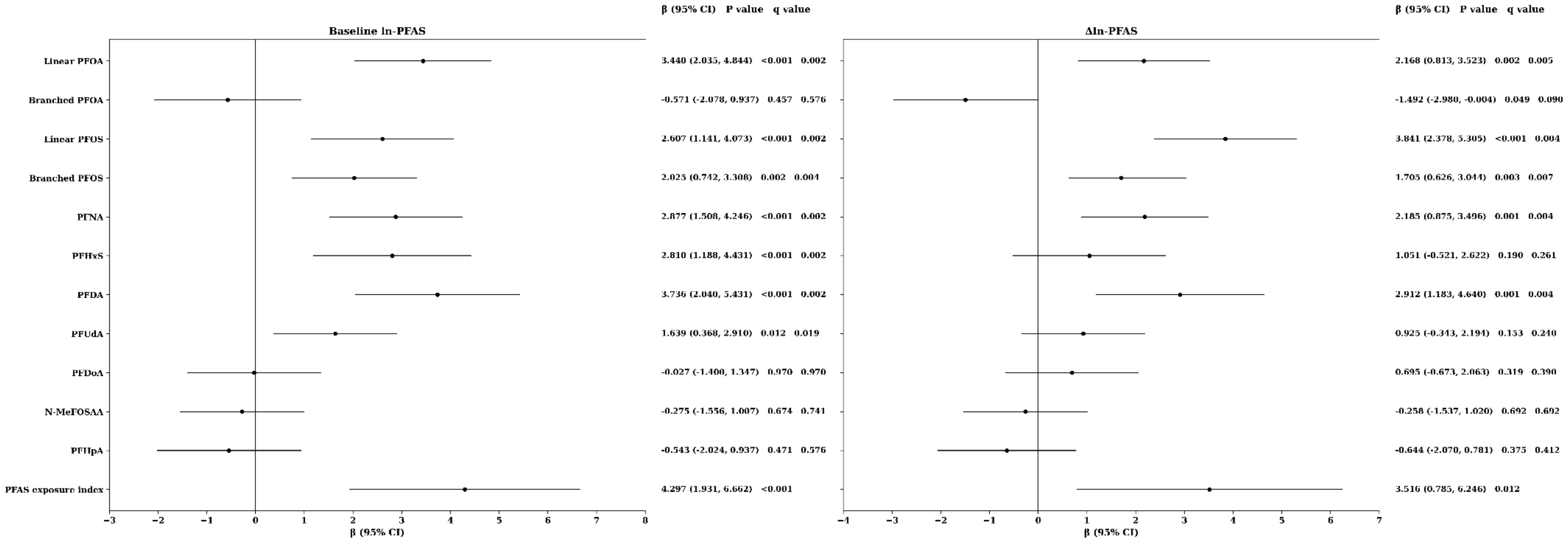

References:

1. Wei, J.N.; Sung, F.C.; Lin, C.C.; Lin, R.S.; Chiang, C.C.; Chuang, L.M. National surveillance for type 2 diabetes mellitus in Taiwanese children. *Jama* **2003**, *290*, 1345-1350, doi:10.1001/jama.290.10.1345.
2. Lin, C.Y.; Wen, L.L.; Lin, L.Y.; Wen, T.W.; Lien, G.W.; Hsu, S.H.; Chien, K.L.; Liao, C.C.; Sung, F.C.; Chen, P.C.; et al. The associations between serum perfluorinated chemicals and thyroid function in adolescents and young adults. *J Hazard Mater* **2013**, *244-245*, 637-644, doi:10.1016/j.jhazmat.2012.10.049.
3. Chen, C.W.; Tang, S.Y.; Hwang, J.S.; Chan, C.C.; Hsu, C.C.; Lin, C.Y.; Su, T.C. Association between Levels of Urine Di-(2-ethylhexyl)phthalate Metabolites and Heart Rate Variability in Young Adults. *Toxics* **2021**, *9*, doi:10.3390/toxics9120351.
